# Kinetic Analysis of the Thermal Degradation of Recycled Acrylonitrile-Butadiene-Styrene by non-Isothermal Thermogravimetry

**DOI:** 10.3390/polym11020281

**Published:** 2019-02-07

**Authors:** Rafael Balart, David Garcia-Sanoguera, Luis Quiles-Carrillo, Nestor Montanes, Sergio Torres-Giner

**Affiliations:** 1 Technological Institute of Materials (ITM), Universitat Politècnica de València (UPV), Plaza Ferrándiz y Carbonell 1, 03801 Alcoy, Spain; rbalart@mcm.upv.es (R.B.); dagarsa@dimm.upv.es (D.G.-S.); nesmonmu@upvnet.upv.es (N.M.); 2 Novel Materials and Nanotechnology Group, Institute of Agrochemistry and Food Technology (IATA), Spanish National Research Council (CSIC), Calle Catedrático Agustín Escardino Benlloch 7, Paterna, 46980 Valencia, Spain; storresginer@iata.csic.es

**Keywords:** thermal degradation, acrylonitrile-butadiene-styrene (ABS), thermogravimetric analysis (TGA), model free kinetics (MFK), combined kinetic analysis (CKA)

## Abstract

This work presents an in-depth kinetic study of the thermal degradation of recycled acrylonitrile-butadiene-styrene (ABS) polymer. Non-isothermal thermogravimetric analysis (TGA) data in nitrogen atmosphere at different heating rates comprised between 2 and 30 K min^−1^ were used to obtain the apparent activation energy (*E_a_*) of the thermal degradation process of ABS by isoconversional (differential and integral) model-free methods. Among others, the differential Friedman method was used. Regarding integral methods, several methods with different approximations of the temperature integral were used, which gave different accuracies in *E_a_*. In particular, the Flynn-Wall-Ozawa (FWO), the Kissinger-Akahira-Sunose (KAS), and the Starink methods were used. The results obtained by these methods were compared to the Kissinger method based on peak temperature (*T_m_*) measurements at the maximum degradation rate. Combined Kinetic Analysis (CKA) was also carried out by using a modified expression derived from the general Sestak-Berggren equation with excellent results compared with the previous methods. Isoconversional methods revealed negligible variation of *E_a_* with the conversion. Furthermore, the reaction model was assessed by calculating the characteristic y(α) and z(α) functions and comparing them with some master plots, resulting in a *n^th^* order reaction model with *n* = 1.4950, which allowed calculating the pre-exponential factor (*A*) of the Arrhenius constant. The results showed that *E_a_* of the thermal degradation of ABS was 163.3 kJ mol^−1^, while ln *A* was 27.5410 (*A* in min^−1^). The predicted values obtained by integration of the general kinetic expression with the calculated kinetic triplet were in full agreement with the experimental data, thus giving evidence of the accuracy of the obtained kinetic parameters.

## 1. Introduction

Thermal degradation of polymers is a topic of great relevance. On one hand, due to their high sensitivity to temperature, degradation or aging at moderate temperatures is responsible for a dramatic loss in overall performance [1,2]. On the other hand, thermal degradation at high temperatures, studied by thermogravimetric analysis (TGA), gives a sound idea of the thermal stability of polymers and composites [3,4]. In addition to conventional analytical techniques, theoretical studies are being conducted in order to elucidate the main parameters governing the thermal degradation of polymers [5].

Acrylonitrile-butadiene-styrene (ABS) is a commodity plastic widely used at the industrial level due to an excellent combination of processability, chemical and light resistance, balanced mechanical properties, and a competitive cost. In addition, ABS is recyclable and compatible with most styrenic polymers and some technical polymers, such as polycarbonate (PC) and polyamides (PAs) [6,7,8]. All these features make ABS the optimal selection for a wide variety of applications, such as electric and electronic equipment, cases and components for electrical appliances, parts and components for the automotive industry, toys, and more recently, as a base material for 3D printing due to the moderate processing temperatures and the excellent surface finish that can be accomplished [9]. ABS consists of a poly(acrylonitrile-*co*-styrene) (SAN) matrix, in which a rubber phase of poly(butadiene) (BR) is finely dispersed and partially grafted, leading to a drop-like structure that positively contributes to increasing toughness [10].

Regarding its thermal stability, as with many other polymers, ABS is highly sensitive to thermo-oxidative processes [11,12]. In addition, at moderate temperatures in the range of 220–240 °C, it undergoes some cross-linking phenomena due to the presence of C=C double bonds in the BR phase [13]. As reported by Polli et al. [14], different analysis of the thermal degradation of ABS can be found in the literature using different thermal programs and atmospheres [15]. As is generally established, degradation of ABS, as similar to other vinyl polymers, proceeds by end-chain and random-chain scissions [14,16,17,18]. In this sense, Perez-Maqueda et al. [19] reported the scission mechanism of atactic poly(styrene) (PS) and validated a method that describes accurately the polymer chain breakage and evaporation of the broken sub-units as a function of the reaction fraction. Despite the complexity of the degradation process, degradation of ABS typically occurs in a single-step process. It has been reported that ABS degradation occurs in several overlapped processes, one corresponding to the styrenic phase and the other corresponding to the butadiene component as validated by Fourier-transform infrared (FTIR) spectroscopy. Each of these processes is governed by an activation energy. Nevertheless, in dynamic TG with constant heating rate, it is impossible to separate them. As it has been reported Luda di Cortemiglia et al. [20], the SAN phase of ABS degrades in a single volatilization process with a maximum rate at 425 °C, and chain fragments, NH_3_, HCN, and aromatic compounds are generated. With regard to the butadiene phase, it degrades at higher temperatures in a two-step process. The first stage represents about 20% weight loss and involves formation of chain fragments and condensable gaseous hydrocarbons, while the second stage is related to the major weight loss and also non condensable gases such as H_2_ and CH_4_ are evolved. These processes occur as a single weight loss step in TGA of ABS, thus indicating it can be analyzed as an overall degradation process with one “apparent” activation energy. The apparent activation energy, *E_a_* that has been reported in the literature cover the range comprised between 134 to 190 kJ mol^−1^ [21]. In a previous work, our research group reported *E_a_* values for the thermal degradation of recycled ABS in the 140–160 kJ mol^−1^ range, depending on the selected kinetic method [22]. The autocatalytic reaction model was proposed as the best model since it gave good accuracy between the experimental data and the values predicted by this reaction model, showing values of *E_a_* = 163.14 ± 3.51 kJ mol^−1^, ln *A* = 28.0543 ± 0.5778 (*A* in min^−1^), and *f(α)* = *α^m^*(1−*α*)*^n^* with *n* = 1.7157 ± 0.2970 and *m* = 0.56 ± 0.1823.

This work originally presents a systematic approach to the kinetic study of the thermal degradation of recycled ABS polymer under nitrogen atmosphere by using several model-free kinetic methods (MFK) with their corresponding accuracies and corrections. Moreover, the usefulness of the Combined Kinetic Analysis (CKA) methodology is assessed by comparing the obtained *E_a_* values with those obtained with conventional isoconversional model-free kinetic methods. Finally, the work deals with the elucidation of the reaction model *f*(α), by calculating the corresponding y(α) and z(α) functions and comparing them with some master curve plots.

## 2. Theoretical Background

Kinetic studies are based on the following general kinetic expression:

(1) $$\frac{d\alpha }{dt}=\;k\;f(\alpha )$$

This expression indicates that the reaction or conversion rate (dα/d*t*) is directly related to the rate or kinetic constant (*k*), and a function of the conversion f(α), which takes several forms, depending on the main mechanism, and subsequently, is representative for the reaction model. The rate constant (*k*) is temperature-dependent and it is assumed to follow the Arrhenius equation in thermally activated reactions in the solid state (Equation (2)):

(2) $${k(T)\;=\;A\;\exp(-E}_{a}/RT)$$

where *A* (in min^−1^) corresponds to the pre-exponential or frequency factor, *E_a_* (in J mol^−1^) stands for the apparent activation energy, *R* is the gas constant (8.314 J mol^−1^ K^−1^), and *T* is the absolute temperature (in K).

By combining Equations (1) and (2), the following expression is obtained:

(3) $$\frac{d\alpha }{dt}{\;=\;A\;\exp(-E}_{a}/RT)\;f(\alpha )$$

As Equation (3) suggests kinetic analysis assists on determining the kinetic triplet: *A*, *E_a_*, f(α).

### 2.1. Isoconversional Methods

Isoconversional methods have been extensively used to estimate E_a_ accurately by using differential or integral methods (in either isothermal or non-isothermal conditions). Differential methods are based on Equation (3), which can be used for whatever thermal program. This time-domain expression can be converted into a temperature-domain by assuming the heating rate (*β*).

(4) $$\beta \;=\;\frac{dT}{dt}$$

Then, by substituting Equation (4) in Equation (3) yields Equation (5). This temperature-dependent equation is applicable to dynamic thermal programs.

(5) $$\beta \frac{d\alpha }{dT}\;=\;A\;\exp(-E_{a}/RT)\;f(\alpha )$$

Equation (5) can also be represented in its integral form to give Equation (6), which is the base for the integral methods.

(6) $$\int_{0}^{\alpha }\frac{d\alpha }{f(\alpha )}=\frac{A}{\beta }\int_{T_{0}}^{T}{\exp(-E}_{a}/RT)\;dT$$

Isoconversional methods derive from the basic isoconversional principle that assumes that the reaction rate at a constant conversion (α*_c_*) is only dependent on temperature. By taking natural logarithms on both sides of Equation (1), it yields:

(7) $$\ln\frac{d\alpha }{dt}\;=\;\ln\;k(T)\;+\;\ln\;f(\alpha )$$

Then, by taking partial derivatives with respect to (1/*T*) on Equation (7) at a constant α = α*_i_*, the following expression is obtained:

(8) $${\left[\frac{\partial \ln\left(\frac{d\alpha }{dt}\right)}{\partial {\;T\;}^{-1}}\right]}_{\alpha_{i}}=\;{\left[\frac{\partial \ln\;k(T)}{\partial {\;T\;}^{-1}}\right]}_{\alpha_{i}}+{\left[\frac{\partial \ln\;f(\alpha )}{\partial {\;T\;}^{-1}}\right]}_{\alpha_{i}}$$

In isoconversional conditions, at a constant α value α*_i_*, its corresponding f(α) value is also constant, and therefore, the second term on the right-hand side of Equation (8) equals zero. The first term on the right-hand side of Equation (8) can be obtained by taking the partial derivative of the natural logarithmic form of Equation (2) with respect to 1/*T*. Therefore, the following expression can be obtained:

(9) $${\left[\frac{\partial \ln\;k(T)}{\partial {\;T\;}^{-1}}\right]}_{\alpha_{i}}=\;{\left[\frac{\partial {\ln\;A\;\exp(-E}_{a}/RT)}{\partial {\;T\;}^{-1}}\right]}_{\alpha_{i}}=\;\frac{{-E}_{{a,\alpha }_{i}}}{R}$$

Then, by combining Equation (9) and Equation (8), the isoconversional methods are based on the fact that it is possible to obtain an estimation of *E_a_* (at a particular α*_i_* = constant) without assuming any reaction model, as shown in Equation (10). Thus, these methods are habitually called Model-Free Kinetic methods (MFK). These methods are widely used to study the apparent activation energy of thermal activated processes (degradation and cross-linking) [23].

(10) $${\left[\frac{\partial \ln\left(\frac{d\alpha }{dt}\right)}{\partial {\;T\;}^{-1}}\right]}_{\alpha_{i}}=\;\frac{{-E}_{{a,\alpha }_{i}}}{R}$$

Although isoconversional methods can give quite accurate values of *E_a_* by using differential methods, such as Friedman, or integral methods, such as the Flynn-Wall-Ozawa (FWO), Kissinger-Akahira-Sunose (KAS), and Starink, among others, no additional information is obtained about the reaction model and further methods are then required.

#### 2.1.1. Differential Isoconversional Methods

The Friedman method is a differential method based on Equation (5). By taking natural logarithms and re-arranging terms, Equation (11) is obtained, which suggests that a plot of $\ln\left(\beta \frac{d\alpha }{dT}\right)$ against 1/*T*, at a constant α, for different heating rates β, gives a linear plot whose slope is $\frac{E_{a}}{R}$. As f(α) is constant at a constant α value, and *A* is also a constant, this approach can give an accurate value of *E_a_* without assuming any particular reaction model.

(11) $$\ln\left(\beta \frac{d\alpha }{dT}\right)\;=\;\ln\;A\;f(\alpha )\;-\;\frac{E_{a}}{RT}$$

#### 2.1.2. Integral Isoconversional Methods

Integral methods are based on the integration of Equation (6) to give the integral form, g(α) of the reaction model, f(α).

(12) $$g(\alpha )\;=\int_{0}^{\alpha }\frac{d\alpha }{f(\alpha )}=\frac{A}{\beta }\int_{T_{0}}^{T}\exp(-E_{a}/RT)\;dT$$

The temperature integral (right-hand side) does not have an analytical solution, so that if we consider the reduced temperature as *x* = *E_a_*/*RT*, a polynomial *P(x)* must be obtained by numerical methods.

(13) $$g(\alpha )\;=\;\frac{{AE}_{a}}{\beta R}\;P(x)$$

As indicated previously, by taking *x* = *E_a_*/*RT* as the reduced temperature, a series expansion can give different approximations of *P(x)*. Asymptotic expansions after a single integration-by-parts are interesting approximations of *P(x)* [24].

(14) $$\;P(x)\;=\;\frac{e^{-x}}{x^{2}}\;\left[1+\frac{2\text{!}}{-x}+\frac{3\text{!}}{{\left(-x\right)}^{2}}+\frac{4\text{!}}{{\left(-x\right)}^{3}}+\text{…}\right]$$

Another asymptotic expansion series is that proposed by Schlomilch, as shown in Equation (15):

(15) $$P(x)\;=\frac{e^{-x}}{x(1+x)}\;\left[1-\frac{1}{\left(2+x\right)}+\frac{2}{\left(2+x)(3+x\right)}-\frac{4}{\left(2+x)(3+x)(4+x\right)}+\text{…}\right]$$

Or the Lyon’s expansion shown in Equation (16) [25]:

(16) $$P(x)\;=\frac{e^{-x}}{x(2+x)}\;\left[1+\frac{2}{x(2+x)}-\frac{8}{{x(2+x)}^{2}}{+\;O(x}^{-4})\;+\text{…}\right]$$

There are several integral methods which use different solutions of the temperature integral, such as the Broido method [26]. The Flynn-Wall-Ozawa (FWO) method allows estimating the apparent activation energies (*E_a_*) over the entire α range from TGA data at different heating rates (β). This method is widely used because it provides an accurate value of *E_a_* for a particular α (isoconversional) value. The FWO method is an integral method that uses the Doyle’s approximation of the temperature integral *P(x)*. The empirical equation proposed by Doyle for the temperature integral is shown in Equation (17) [27,28,29,30]:

(17) $$P(x)\;={7.03\;\times \;10}^{-3}{\;e\;}^{x\;B(x)}$$

where *B(x)* is comprised between −1.195 and −1.034 over the *x* domain. Usually, an average value of −1.052 is used for integral methods for *x* values comprised between 10 and 60 with a relatively low error. By substituting Equation (17) in Equation (13), it yields:

(18) $$g(x)\;=\;\frac{{AE}_{a}}{\beta R}{7.03\;\times \;10}^{-3}e^{-1.052x}$$

By taking natural logarithms on both sides of the equality and rearranging terms, Equation (19) is obtained. This equation is the base of the FWO integral method:

(19) $$\begin{array}{c} \ln(\beta )=\ln\left(\frac{{AE}_{a}}{R}\right)-\ln(g(\alpha ))\;-\;4.9575\;-\;1.052\frac{E_{a}}{RT}\\ \ln(\beta )=\;\mathrm{Constant}\;-\;1.052\frac{E_{a}}{RT}\\ \mathrm{or}\;\log(\beta )=\;\mathrm{Constant}\;-\;0.4567\frac{E_{a}}{RT} \end{array}$$

As it can be seen from Equation (19), a plot of log(β) versus *1/T* (or ln(β) versus *1/T*) allows a linear fit, whose slope is directly related to the *E_a_* at that particular α value [31].

Different approximations give different expressions, which can be useful to estimate the values of *E_a_*. The FWO method is not the most accurate, as it makes use of a quite essential approximation of *P(x)*. Among others, the Kissinger-Akahira-Sunose method (KAS) employs the Murray and White approximation of *P(x)* as indicated in Equation (20), which gives more accurate results:

(20) $$P(x)\;≅\;\frac{e^{-x}}{x^{2}}$$

By using the KAS method (Equation (21)), a plot representation of ln$\left(\frac{\beta }{T^{\;2}}\right)$ versus 1/*T* for a particular conversion α, gives a straight line with a slope consisting on *E_a_/R* [32].

(21) $$\begin{array}{c} \ln\left(\frac{\beta }{T^{\;2}}\right)=\;\ln\left(\frac{AR}{E_{a}}\right)-\ln\;(g(\alpha ))-\;\frac{E_{a}}{RT}\\ \ln\left(\frac{\beta }{T^{\;2}}\right)=\;\mathrm{Constant}\;-\;\frac{E_{a}}{RT} \end{array}$$

Another approximation is that given by Starink [24] in Equation (22), which allows an even more accurate estimation of *E_a_*.

(22) $$\ln\left(\frac{\beta }{T^{1.92}}\right)=\;\mathrm{Constant}\;-\;1.0008\frac{E_{a}}{RT}$$

As one can realize, Equation (19), Equation (21), and Equation (22) show similar mathematical structures as Starink [24] already demonstrated, thus leading to a generalized expression:

(23) $$\ln\left(\frac{\beta }{T^{\;B}}\right)\;=\;\mathrm{Constant}\;-\;C\frac{E_{a}}{RT}$$

where *B* and *C* are experimental values related to a particular approximation of the temperature integral, so that Doyle’s approximation considers *B* = 0 and *C* = 1.052. The Murray and White approximation leads to *B* and *C* values of 2 and 1, respectively.

### 2.2. Combined Kinetic Analysis (CKA)

The above-mentioned methods allow estimating the apparent activation energies without any assumption of the reaction model. As it has been described previously, kinetic analysis offers several tools to determine the so-called kinetic triplet, that is, *E_a_*, *A*, and *f(α)*. One interesting approach to get more information and simultaneously obtain *E_a_* and *A* is the Combined Kinetic Analysis (CKA). Equation (3) can be written in the following form by taking natural logarithms on both sides and rearranging terms:

(24) $$\ln\left(\frac{\beta \;(\frac{d\alpha }{dT})}{f(\alpha )}\right)\;=\;\ln\;A\;-\;\frac{E_{a}}{RT}$$

By choosing the appropriate kinetic model, *f*(α), a plot of the left-hand side of Equation (24) versus 1/*T*, allows determining *E_a_* from the slope of the linear fit while the pre-exponential factor, *A*, can be obtained from the intercept once *E_a_* has been obtained. Obviously, the quality of the linear fit will depend on the selected reaction model, *f*(α). Table 1 summarizes several kinetic models with their typical *f*(α) functions and their corresponding integral forms, *g*(α).

In addition to the correct selection of the kinetic model, experimental data can deviate from the theoretical kinetic models due to sample size, geometry, shape, and so on. These drawbacks can be overcome or minimized by the procedure described by Perez-Maqueda et al. [33], which suggests the use of a generic equation derived from the generalized Sestak-Berggren equation shown in Equation (25) [34]. Depending on the combination of the superscripts, *m*, *n* and *p*, Equation (25) can represent almost any reaction model.

(25) $${f(\alpha )\;=\;\alpha }^{m}{\;(1-\alpha )}^{n}{\left[-\ln(1-\alpha )\right]}^{p}$$

The generalized expression proposed by Perez-Maqueda et al. [33] is shown in Equation (26), and as it has been demonstrated, it can fit most of the typical reaction models by simply adjusting the *c*, *m*, and *n* parameters.

(26) $${f(\alpha )\;=\;c\;\alpha }^{m}{\;(1-\alpha )}^{n}$$

If Equation (26) is included into Equation (24) and the terms are rearranged, it gives Equation (27). An optimization of the Pearson’s linear correlation coefficient can be considered as the objective to obtain the optimum m and n values that allow a linear fit between the left-hand side part of Equation (27) against 1/*T*. Once m and n are obtained, the *E_a_* and ln *cA* values can be obtained from the slope and intercept of the optimum linear fit, respectively, as reported by Sánchez-Jiménez et al. [35].

(27) $$\ln\left(\frac{\beta \;(\frac{d\alpha }{dT})}{\alpha^{m}{\left(1-\alpha \right)}^{n}}\right)=\ln\;c\;A\;-\frac{E_{a}}{RT}$$

### 2.3. Criado and Maqueda Method

Criado-Maqueda methodology is based on the use of master plots corresponding to different reaction models (Table 1). By comparison with experimental data, it is possible to elucidate the reaction model if it is unique, or in some cases, if it is based on two or more consecutive reaction models, depending on the conversion, α. As previously stated, isoconversional models allow estimating the E_a_ in an accurate way but they do not give any information about the reaction model. Once *E_a_* has been obtained, a *y*(α) function can be defined as shown in Equation (28), with *x* = *E_a_*/*RT* representing the reduced temperature:

(28) $$y(\alpha )\;=\frac{\left(\frac{d\alpha }{dt}\right)}{e^{-x}}$$

Substituting Equation (5) in Equation (28) and rearranging terms, it yields Equation (29). Since *A* is a constant value, the plot of the second member in Equation (29) against α should be proportional to *f*(α). Therefore, *y*(α) gives the geometry of *f*(α) and it can be very useful to elucidate the reaction model. It is possible to normalize *y*(α) to enable comparison with the normalized theoretical functions in Table 1.

(29) $$y(\alpha )\;=\left(\frac{\beta \;(\frac{d\alpha }{dT})}{\exp(-E_{a}/RT)}\right)\;=\;A\;f(\alpha )$$

To give more consistency to this method, Criado-Maqueda also proposed the use of a *z*(α)-type function to represent the master curves corresponding to different reaction models that take into account both *f*(α) and its integral form *g*(α). The *z*(α) function is defined in Equation (30), in which π(x) is related to the Arrhenius temperature integral:

(30) $$z(\alpha )\;=\;\frac{\frac{d\alpha }{dt}}{\beta }\pi (x)\;T$$

As observed in Equation (14), Equation (15), and Equation (16), the approximations of the temperature integral are defined by a series of asymptotic functions. Nevertheless, all these equations can be expressed for a particular expansion, as indicated in Equation (31):

(31) $$P(x)\;=\;\frac{e^{-x}}{x}\;\pi (x)$$

Senum and Yang have proposed different approximations of π(x) in a rational form, as summarized in Table 2 [36]. Perez-Maqueda et al. [37] validated the accuracy of these approximations up to eight degrees and they concluded that a π(x) rational approximation with a fourth degree gives an error lower than 10^−5^, therefore the fourth degree is enough for kinetic studies although they have demonstrated increasing accuracy with increasing degree. Table 2 shows the corrected value for the first power x term of the numerator of the fourth degree of 86 instead of 88 as reported by Flynn [38].

Then, by substituting Equation (3) and Equation (31) in Equation (30), the following expression is derived:

(32) z(α) = f(α)·g(α)

As the E_a_ of thermally activated processes can be determined by several non-isothermal tests independently of the reaction mechanism f(α), the theoretical master plots obtained by Equation (32) for different well-established reaction mechanisms, as summarized in Table 2, can be easily obtained. These plots can be very useful in determining the main reaction mechanism or even the existence of overlapped reaction mechanisms, as the z(α) corresponding to experimental results can be calculated by using Equation (33), which is derived from including π(x) from Equation (31) on Equation (20) and considering *x* = *E_a_*/*RT*.

(33) $$z(\alpha )\;=\;\frac{d\alpha }{dt}\frac{E_{a}}{\beta R}\;\exp(\frac{E_{a}}{RT})P(x)$$

Or by taking into account the heating rate, β, Equation (33) can be written as:

(34) $$z(\alpha )\;=\;\frac{d\alpha }{dT}\frac{E_{a}}{R}\;\exp(\frac{E_{a}}{RT})P(x)$$

As in the case of y(α), a comparison of the calculated z(α) from the experimental data using Equation (33) or Equation (34) and the different normalized z(α) master plot curves corresponding to different reaction mechanisms for solid state reactions, as described in Table 2, and calculated by Equation (32), can be very useful to assess the reaction model or a combination of them.

## 3. Materials and Methods

### 3.1. Materials

**Scheme 1:**
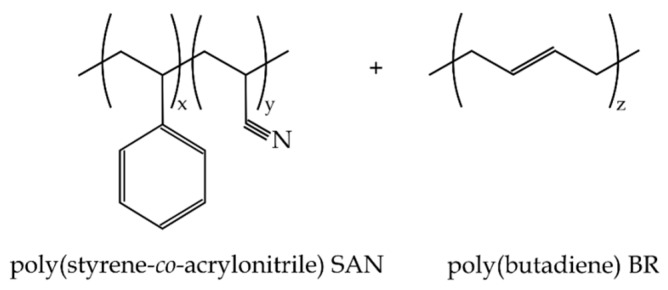
Schematic plot of the acrylonitrile-butadiene-styrene (ABS) structure, mainly composed of poly(styrene-*co*-acrylonitrile) (SAN) and poly(butadiene) rubber (BR).

A recycled acrylonitrile-butadiene-styrene (ABS) obtained from waste electric-electronics equipment (WEEE) was used as the base material for kinetic analysis. This ABS grade is a binary blend of SAN and BR. Its characteristic glass transition temperature (*T*_g_) is located at 104.4 °C. The melt flow index (MFI) is 7.7 g/10 min, measured at 210 °C and a load of 5 kg, and 20.5 g/10 min, measured at 230 °C and a load of 5 kg in a ATF Faar S.p.A. melt flow index (MFI) (Novegro-Tregarezzo, Italy). Scheme 1 shows a plot of the structure of this polymer blend.

### 3.2. Thermal Degradation Analysis

Kinetic analysis of the thermal degradation of recycled ABS was carried out by using dynamic thermogravimetry (TGA). To this end, a TGA/SDTA 851 thermobalance from Mettler-Toledo (Schwerzenbach, Switzerland) was used. An average sample mass of 8–10 mg was subjected to a dynamic program from 30 °C up to 700 °C at different heating rates (2, 5, 10, 15, 20, 25, and 30 K min^−1^) in nitrogen atmosphere with a constant nitrogen flow of 80 mL min^−1^. Nitrogen atmosphere was selected to simulate typical “pyrolysis” conditions, which could be helpful for practical uses, such as energy valorization of plastic wastes. Standard graphite crucibles were used. The conversion α at each temperature was calculated using Equation (35):

(35) $$\alpha \;=\frac{w_{0}\;{-\;w}_{T}}{w_{0}\;{-\;w}_{f}}$$

where w_0_ denotes the initial mass of the sample, *w_f_* stands for the residual mass, and *w_T_* represents the mass at a particular temperature (*T*).

## 4. Results and Discussion

### 4.1. Determination of the Apparent Activation Energy of the Thermal Degradation of ABS

**Figure 1:**
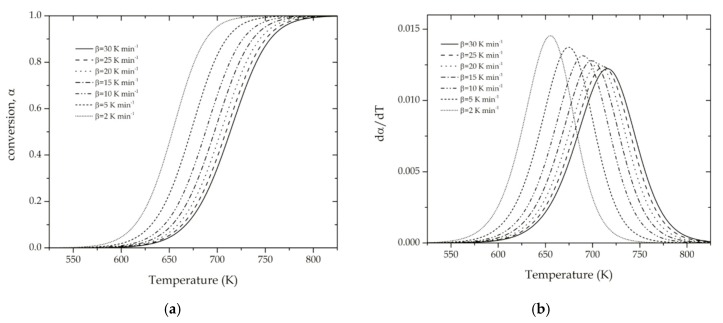
(**a**) Plot of the conversion α vs. temperature for the degradation of acrylonitrile-butadiene-styrene (ABS) at different heating rates, and (**b**) plot of the first derivative of α with regard to temperature (dα/d*T*) vs. temperature.

The typical conversion curves for the degradation of recycled ABS are shown in Figure 1a with varying α. Figure 1b shows the corresponding (dα/d*T*) plots versus *T*, including the characteristic temperatures for the maximum degradation rates (*T*_m_). A simple method to obtain the *E_a_* from a set of experiments at different heating rates is the Kissinger method [32]. This is widely used because of its simplicity, and it is based on the assumption that $\frac{d}{dt}\left(\frac{d\alpha }{dt}\right)=\;0$ at *T_m_*. It is assumed that the conversion at the maximum degradation rate, α*_m_*, is almost constant for a series of heating rates, β. Derivation of Equation (3) gives the following expression:

(36) $$\frac{d}{dt}\left(\frac{d\alpha }{dt}\right)=\;\left\{{A\;\exp(-E}_{a}/RT)\;\frac{E_{a}}{{RT}^{\;2}}\left(\frac{dT}{dt}\right)f(\alpha )\;+\;\left(\frac{d\;f(\alpha )}{d\alpha }\right)\left(\frac{d\alpha }{dt}\right){A\;\exp(-E}_{a}/RT)\right\}$$

Taking into account the definition of the heating rate β, as indicated in Equation (4), arranging terms in Equation (36) and considering the definition of $\left(\frac{d\alpha }{dt}\right)$ in Equation (3), Equation (36) can be written as:

(37) $$\frac{d}{dt}\left(\frac{d\alpha }{dt}\right)=\;\left(\frac{d\alpha }{dt}\right)\left\{\frac{{\beta E}_{a}}{{RT}^{\;2}}\;+\;\left(\frac{d\;f(\alpha )}{d\alpha }\right){A\;\exp(-E}_{a}/RT)\right\}$$

As indicated previously, the Kissinger methods is based on the assumption $\frac{d}{dt}\left(\frac{d\alpha }{dt}\right)=\;0$, therefore, by considering Equation (37) and rearranging terms, the following expressions are obtained:

(38) $$\frac{{\beta E}_{a}}{{RT}_{m}^{\;2}}\;+\;\left(\frac{d\;f(\alpha )}{d\alpha }\right){A\;\exp(-E}_{a}{/RT}_{m})\;=\;0\;$$

(39) $$\frac{\beta }{T_{m}^{\;2}}\;=\;-\frac{AR}{E_{a}}\left(\frac{d\;f(\alpha )}{d\alpha }\right){\exp(-E}_{a}{/RT}_{m})$$

Then, by taking natural logarithms on both sides of Equation (39), it yields:

(40) $$\ln\left(\frac{\beta }{T_{m}^{\;2}}\right)\;=\;\ln\left(\frac{AR}{E_{a}}\right)+\;\ln\left(-\frac{d\;f(\alpha )}{d\alpha }\right)-\frac{E_{a}}{{RT}_{m}}$$

For many reaction models, the second term in the right-hand side of Equation (40) is constant [39], thus suggesting that a simple plot of $\ln\left(\frac{\beta }{T_{m}^{\;2}}\right)$ *versus* 1/*T_m_*, at different heating rates β, yields a linear plot whose slope is −*E_a_*/*R*. If the term $\left(-\frac{d\;f(\alpha )}{d\alpha }\right)$ is dependent on the heating rate, then the linear relationship cannot be inferred, which represents a limitation of this method [40]. Table 3 summarizes the main results obtained in relation to *T_m_* and the conversion for each *T_m_* value (α*_m_*). As it can be seen, the conversion at the peak temperature is almost identical with an average value for the different heating rates of 0.531 ± 0.004.

**Figure 2:**
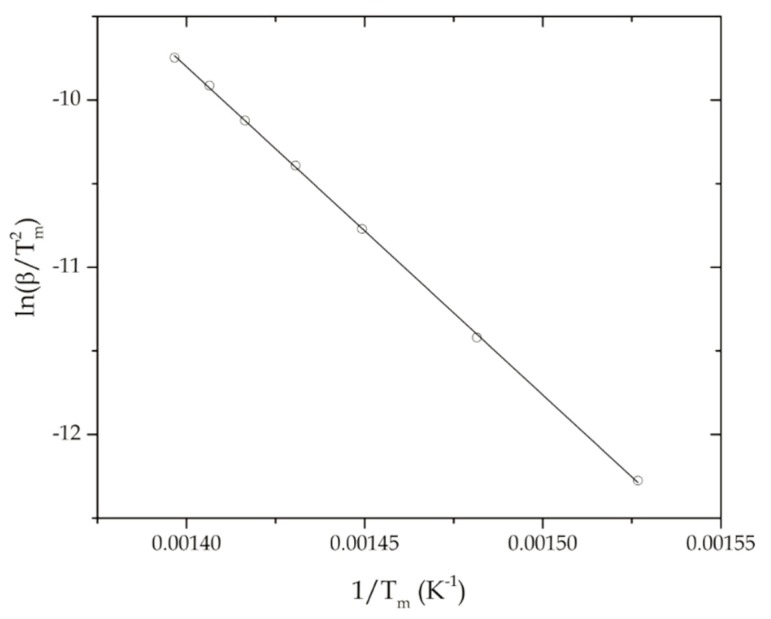
Plot of $\ln\left(\frac{\beta }{T_{m}^{\;2}}\right)$ versus 1/*T_m_* by applying the Kissinger method corresponding to the degradation of recycled acrylonitrile-butadiene-styrene (ABS) at different heating rates β.

As one can observe in Figure 2, the correlation between $\ln\left(\frac{\beta }{T_{m}^{\;2}}\right)$ versus 1/*T_m_* is very accurate and it allows for estimating a single value of *E_a_*. In particular, the *E_a_* value obtained was 163.1±1.0 kJ mol^−1^ with a correlation coefficient *r* = −0.9998. One of the assumptions of this method is that f(α) does not change with α. In fact, it is giving *E_a_* at a particular conversion α*_m_* = 0.531. The Kissinger method gives a reliable value of *E_a_* if α*_m_* does not change in a significant way with the heating rate, β. On the other hand, the systematic error decreases with increase of the reduced temperature *x* = *E_a_*/*RT*. In this particular case, *x* was comprised between 27.40 and 29.95 for the corresponding *T_m_* values. As it has been reported, *x* values higher than 10 lead to an error of less than 5% in *E_a_* [41,42].

On the other hand, due to its simplicity, the Kissinger method can only represent a single-step kinetic process as it is derived from Equation (3). Additional tests are needed to validate the applicability of the Kissinger method. In particular, as indicated by Farjas et al. [43], the peak width of the degradation rate $\frac{d\alpha }{dT}$, is a very sensitive parameter to the existence of multiple transformations. As indicated, all the thermo-analytical curves differ by a time scale factor τ, which can be defined as the inverse of the Arrhenius constant that is shown in Equation (41):

(41) $$\tau \;\equiv \;\frac{1}{k(T)}\;\equiv \;\frac{1}{{A\exp(-E}_{a}/RT)}$$

**Figure 3:**
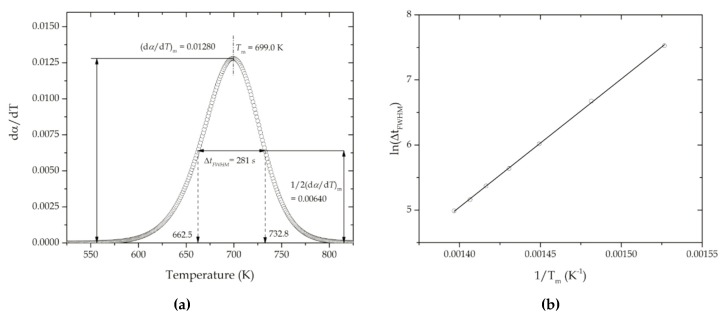
(**a**) Graphical explanation of the time corresponding to the full width at half maximum ($\Delta t_{FWHM}$) for the kinetic degradation of recycled ABS obtained from the $\left(\frac{d\alpha }{dT}\right)$ *vs. T* curve at a constant heating rate of 15 K min^−1^. (**b**) Plot of $\Delta t_{FWHM}$ *vs.* 1/*T_m_* to check the occurrence of a single-step degradation process.

There is a direct relationship between the peak width, measured as the time corresponding to the full width at half maximum (Δ*t_FWHM_*) of the $\left(\frac{d\alpha }{dT}\right)$ curve that is shown in Figure 3a, and the time scale factor corresponding to the peak temperature (*T_m_*), τ*_m_* (Equation (42)).

(42) $$\Delta t_{FWHM}=\;\Delta {t\prime }_{FWHM}{\cdot \tau }_{m}$$

where $\Delta {t\prime }_{FWHM}$ represents the full width at half maximum of a curve obtained when time is normalized to $\tau_{m}$ = 1. Therefore, $\Delta {t\prime }_{FWHM}$ depends only on the reaction model and is a constant value, as reported by Farjas et al. [43] Then, substituting $\tau_{m}$ in Equation (42), applying natural logarithms and rearranging terms, Equation (42) results in Equation (43), which suggests a linear relationship between $\ln\;\Delta t_{FWHM}$ and 1/*T_m_*. If ABS degradation follows a single-step kinetic process, then the *E_a_* value obtained by Equation (43) must be similar or identical to that obtained by the Kissinger method. Otherwise, the assumptions of the Kissinger method would not be appropriate for this particular system.

(43) $$\ln\;\Delta t_{FWHM}=\;\frac{E_{a}}{{RT}_{m}}+\;\ln\;\frac{\Delta {t\prime }_{FWHM}}{A}$$

The *E_a_* value obtained after the linear fit between $\Delta t_{FWHM}$ vs. 1/*T_m_* at different heating rates was 163.7 ± 1.1 kJ mol^−1^, which is in total agreement with the *E_a_* value obtained by applying the Kissinger method. Then, it can be concluded that ABS degradation occurs in a single-step process and the assumptions of the Kissinger method are appropriate, thus giving a reliable *E_a_* value.

**Figure 4:**
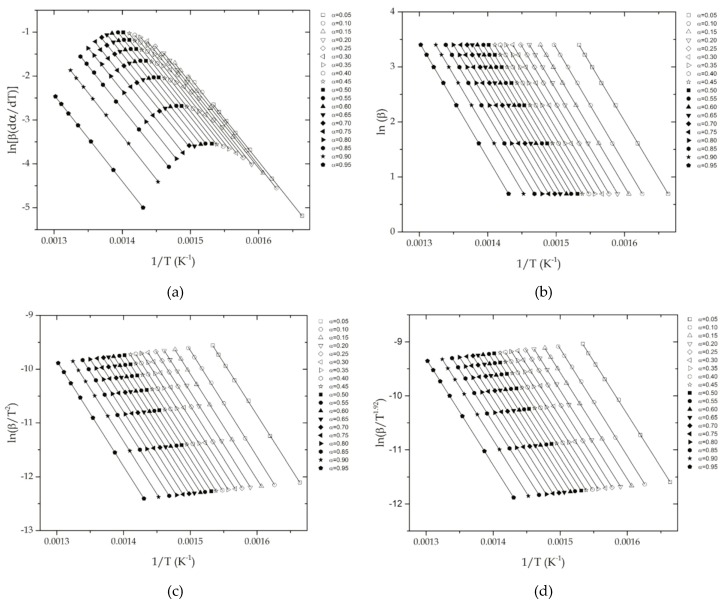
Linear plots corresponding to fitting of experimental data in isoconversional conditions: (**a**) Friedman method showing a plot of $\ln\left(\beta \frac{d\alpha }{dT}\right)$ *vs.* 1/*T*; (**b**) Flynn-Wall-Ozawa (FWO) method showing a plot of ln(β) *vs.* 1/*T*; (**c**) Kissinger-Akahira-Sunose (KAS) method showing a plot of $\ln\left(\frac{\beta }{T^{\;2}}\right)\;vs.\;$ 1/*T*; and (**d**) Starink method showing a plot of $\ln\left(\frac{\beta }{T^{1.92}}\right)$ *vs.* 1/*T*.

Although the premises of the Kissinger method have been checked and the *E_a_* value was accurate, it represents the *E_a_* for a particular conversion α*_m_* = 0.531. To evaluate the possible changes in the reaction model during the conversion, isoconversional methods were applied at different conversion values, since they can give much more information about the variability (or not) of *E_a_* with the conversion, α. Figure 4 shows the typical linear fit plots corresponding to different isoconversional methods, both differential and integral. For all the four methods, isoconversional values ranged between α = 0.05 and α = 0.95 at an increasing step of Δα = 0.05. Figure 4a shows the linear fits in the above mentioned α range according to the Friedman method, which is a differential method. In contrast to the integral methods, which rely on some approximation of the temperature integral, the Friedman method seems to be more accurate, since it does not need any approximation. By using the Friedman method (see Equation (11)), the slope of the linear fit corresponds to −*E_a_*/*R*. The average *E_a_* obtained by the Friedman method was 162.9 ± 1.3 kJ mol^−1^, which indicates a very low variability of *E_a_* with α. All the Pearson’s correlation coefficients for the linear fits were at least −0.9995. This narrow value of *E_a_*, however, does not necessarily means that ABS degradation is a simple degradation process. In fact, it is a complex process consisting of numerous overlapped consecutive and competitive reactions, though the overall process can be considered as a single-step process with unique leading “apparent” activation energy.

As previously indicated, differential methods do not need any approximation, so that in the first instance they could seem more accurate than integral methods. Nevertheless, the application of these methods to integral data, such as those obtained by TGA, involves calculating the first derivative by numerical methods, with the corresponding inaccuracy being due to its noisy signal. With regard to integral methods, although they use more or less accurate approximations of the temperature integral, they can help in obtaining reliable values for *E_a_* [44]. As indicated by Nikolaidis et al. [45], application of differential and integral methods usually gives differences in *E_a_* but the integral methods become more accurate as the accuracy of the temperature integral increases. The FWO is one of the widely used integral methods, though it uses a quite rude approximation of the temperature integral. As indicated in Equation (19), by using the FWO method, the linear fit of ln(β) *vs.* 1/*T* yields a straight line whose slope is −1.052 *E_a_*/*R*. Using this equation, the average *E_a_* obtained was 165.7 ± 0.8 kJ mol^−1^ (with good Pearson’s correlation factor *r* of at least −0.9998), which is in total accordance with the previous results. As the FWO method uses a quite rude approximation of the temperature integral, different corrections have been proposed [24,46,47,48]. As indicated by Venkatesh et al. [49], the Doyle’s approximation can lead to an error of more than 10% in the *E_a_* values. If *E_a_* is independent of the conversion α, the FWO method gives similar values to those obtained by the Friedman method. As pointed out by Flynn [48], the error related to the Doyle’s approximation is less than 1% for reduced temperature values, *x* = *E_a_*/*RT*, comprised between 21 and 81. The reduced temperature values *x* for the average temperatures corresponding to an isoconversional α value at different heating rates were between 27 (α_iso_ = 0.95) and 31 (α_iso_ = 0.05), so that the expected error in the FWO method was less than 1%. By applying the corrections described by Flynn [48], the corrected activation energy *E_a_* was 163.4 ± 0.8 kJ mol^−1^, which is close to the value given by the Friedman method. The KAS method (Figure 4c) uses a more accurate solution for the temperature integral, thus, in a first instance it gives more accurate values. In a similar way to the above-mentioned methods, the KAS gave an almost independent *E_a_* value with the conversion α, with an average value of *E_a_* of 163.0 ± 0.8 kJ mol^−1^, with *r* values close to −0.9999 for all isoconversional α values considered. The value obtained for *E_a_* is in total agreement with the previous values reported by the Friedman method and it is almost the same value than that obtained by using the Starink method, which uses an even better approximation for the temperature integral. In fact, the linear fits using the Starink method (Figure 4d) resulted in an average value of *E_a_* = 163.3 ± 0.8 kJ mol^−1^ (*r* values equal or better than −0.9998), thus giving more consistency to the previous differential and integral methods. In addition, the non-dependency observed for *E_a_* with the conversion suggests that although the degradation reactions could be quite complex, the overall degradation takes place in a single-step process, and then all the assumptions that have been previously made with the different methods lead to coherent values.

It is not easy to elucidate the reaction model by using isoconversional methods. Huang [50] used the Coats and Redfern integral method with different *g*(α) functions and this allowed an approach to the reaction model. Nevertheless, in the last years, with the development of advanced computational methods, new approaches have been proposed. One of them is the so-called Combined Kinetic Analysis (CKA) proposed by Criado-Maqueda [33,35,51,52,53,54,55]. This method makes it possible to obtain the *E_a_* value by assuming a general expression for *f*(α) and maximizing the linear fit between $\ln\left(\frac{\beta \;(\frac{d\alpha }{dT})}{\alpha^{m}{\left(1-\alpha \right)}^{n}}\right)$ as a function of 1/*T* as indicated in Equation (27). As the slope of these linear fits is negative, then the goal has been minimizing the Pearson’s correlation factor (*r*) by varying the *n* and *m* exponents. The results obtained by using this optimization process are summarized in Table 4. It is worthy to note that the linear fit has been applied in a wide conversion range, comprised between α = 0.01 and α = 0.99, and the optimization procedure was possible by using the “minimize” function in MathCad 15 from PTC (Boston, MA, USA).

**Figure 5:**
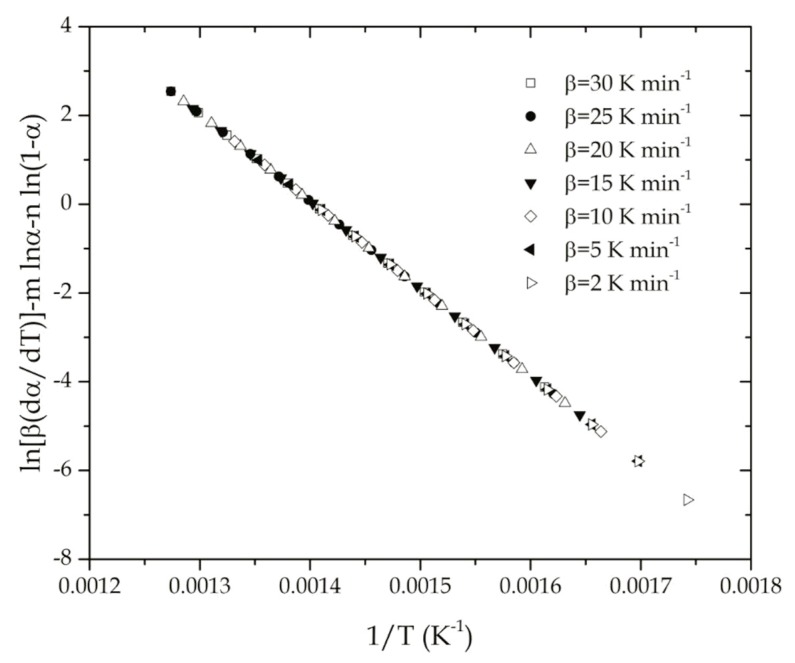
Linear fits corresponding to the Combined Kinetic Analysis (CKA) of the thermal degradation of recycled acrylonitrile-butadiene-styrene (ABS) at different heating rates, β, using Equation (27).

Figure 5 shows the typical plots, which suggest an excellent linear fit (*r* = −1.0000) between the combination of experimental data $\beta (\frac{d\alpha }{dT})$ and a generic mathematical expression that covers almost all potential reaction models in thermally activated processes (${c\alpha }^{m}{\left(1-\alpha \right)}^{n}$). It is worthy to note that the average *E_a_* obtained by Combined Kinetic Analysis (CKA) is very similar to those obtained with the previous methods (*E_a_* = 163.7 ± 0.2 kJ mol^−1^). Although the use of this modified Sestak-Berggren equation (Equation (26)) is only considered for mathematical purposes, the obtained results for *n* = 1.4926 ± 0.0005 and *m* = 0.0032 ± 0.0009 suggest that the (1−α)*^n^* term of this mathematical expression contributes in a major extent than the $\alpha^{m}$ term. Therefore, although this method is based only on a mathematical expression that covers all the possible (or a wide variety of) reaction models, it can also suggest the reaction model for the thermal degradation of recycled ABS. One can considerer the *n^th^* reaction model as the main reaction model. The extremely low SD obtained for *E_a_*, is also representative for its independency from the heating rate, β, which indicates increased consistency to the assumptions of the model, and subsequently, the obtained results are representative. In fact, the *E_a_* value given by the CKA is within the same range of all isoconversional methods and the Kissinger method as well.

### 4.2. Determination of the Reaction Model of the Thermal Degradation of ABS

**Figure 6:**
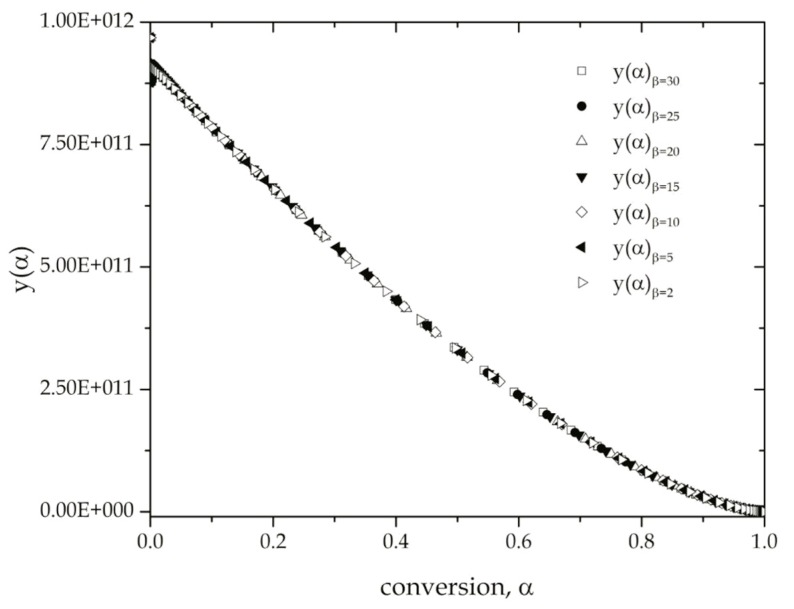
Calculated y(α) functions for the thermal degradation of recycled acrylonitrile-butadiene-styrene (ABS) at different heating rates as a function of the conversion, α, using Equation (28).

**Figure 7:**
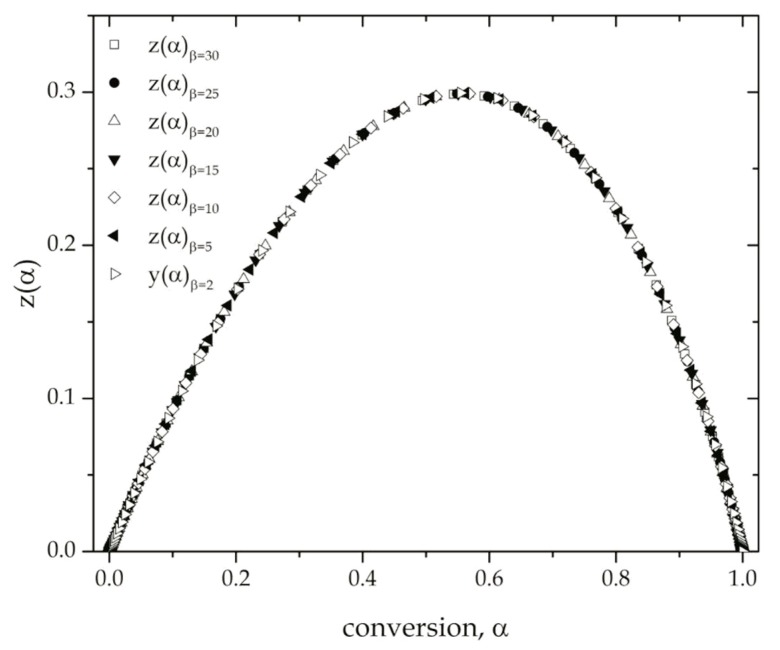
Calculated z(α) functions for the thermal degradation of recycled acrylonitrile-butadiene-styrene (ABS) at different heating rates β, as a function of the conversion, α, using Equation (34).

As previously indicated, all the above-mentioned methods give coherent and almost identical *E_a_* values from different approaches. Nevertheless, one step beyond includes determining the remaining kinetic parameters to give a complete kinetic triplet (*E_a_*, *A*, *f*(α)) that will be helpful to predict the thermal degradation of recycled ABS and also to check the accuracy between the experimental data and the values predicted by the given kinetic model. To this end, the Criado-Maqueda method [17,56,57,58] was employed. This considers a comparison of the calculated y(α) and z(α) plots with several well-known reaction models. For this purpose, y(α) and z(α) were calculated as indicated in Equations (28) and (34), respectively. These functions have been calculated for each heating rate and are shown in Figure 6 and Figure 7. As it can be seen in Figure 6, corresponding to y(α) curve plots, the maximum $\alpha_{M}$ for this plot is close to 0 and its geometry suggests a *n^th^* order reaction model. Moreover, the different y(α) curves for the different heating rates are almost coincident; y(α) suggests a *n^th^* reaction model and the plot of z(α) corroborates this, as can be seen in Figure 7. For a *n^th^* order reaction model, the maximum for the z(α) plot, $\alpha_{p}^{\infty }$ is located at ${1-n}^{\left(\frac{1}{1-n}\right)}$ [59], which resulted in an average theoretical *n* value of 1.4810 by using the values described in Table 5.

**Figure 8:**
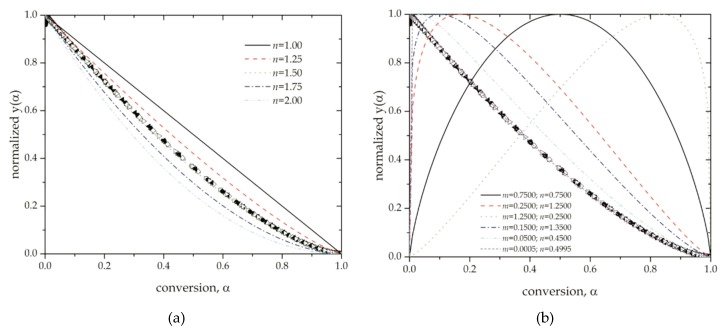
Comparison of calculated y(α) plots with the master plots corresponding to (**a**) the *n^th^* order reaction model and (**b**) the autocatalytic reaction model, with different exponent parameters. Normalized y(α) functions for different heating rates β: □ 30 K min^−1^, ● 25 K min^−1^, △ 20 K min^−1^, ▼ 15 K min^−1^, ◇ 10 K min^−1^, ◀ 5 K min^−1^, and ▻ 2 K min^−1^.

By assuming a *n^th^* order reaction model, with *f*(α) = (1−α)*^n^*, both the reaction order and ln *A* values can be simultaneously obtained by linear fit of ln y(α) as a function of ln(1−α). In fact, the slope of this linear fit corresponds to the reaction order *n*, while the slope represents ln *A*. In addition to the *n^th^* reaction order, the conditions for an autocatalytic process are covered by the calculated y(α) and z(α) plots with $\alpha_{M}$ > 0 and $\alpha_{p}^{\infty }$ > $\alpha_{M}$, using the characteristic peak values shown in Table 5. For this reason, the *n* and *m* values of the autocatalytic reaction model were calculated by the non-linear fitting of ln y(α) as a function of the conversion α (curve fitting to *k* + *m* ln α + *n* ln(1−α)). Table 6 summarizes the results obtained for both reaction models and their corresponding fitting processes. Application of both reaction models suggests that the *n^th^* order reaction model is the best. In fact, when the autocatalytic reaction model is applied, the autocatalytic exponent, *m* was very low. This can be clearly observed in Figure 8, which shows a comparison of the calculated y(α) functions for different heating rates with different normalized master plots. Figure 8a shows a comparison with typical master plots of the *n^th^* order reaction model with different *n* values from 1.00 (linear) to 2.00 (curved line). The master plot for *n* = 1.5 gave high coincidence with the calculated y(α) plots. With regard to the autocatalytic master plots, Figure 8b shows different exponent combinations (with *n* + *m* = 1.5). As one can observe, if *n* = *m*, the maximum is located at a conversion α of 0.5. If *m* > *n*, the maximum is moved to higher α values thus indicating prevalence of the autocatalytic term. On the contrary, if *m* < *n*, the maximum is displaced to lower α values, thus indicating a minor effect of the autocatalytic contribution. As *m* becomes lower (e.g., *n* = 1.4995, *m* = 0.005), the characteristic plot looks like the typical master plot of the *n^th^* order reaction model.

**Figure 9:**
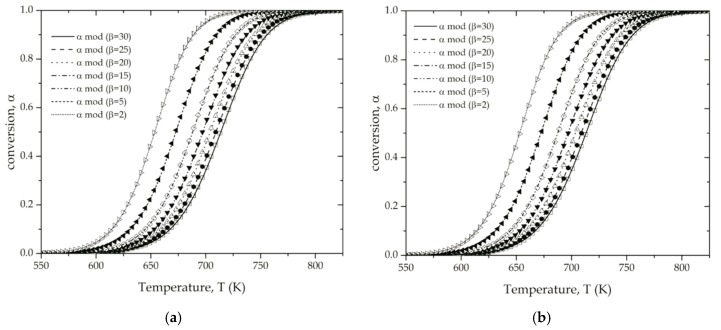
Comparison of experimental data (symbols) and model prediction (lines) with the calculated kinetic triplet for the thermal degradation of recycled acrylonitrile-butadiene-styrene (ABS) at different heating rates β: □ 30 K min^−1^, ● 25 K min^−1^, △ 20 K min^−1^,▼ 15 K min^−1^, ◇ 10 K min^−1^, ◀ 5 K min^−1^, and ▻ 2 K min^−1^. Considering different reaction models (**a**) *n^th^* order and (**b**) autocatalytic.

Once the kinetic triplet was obtained, integration of the conversion rate with the corresponding parameters was carried out and its results are shown in Figure 9. The integration was carried out in Mathcad using the fourth-order Runge-Kutta fixed-step method for both *n^th^* order and autocatalytic model. Figure 9a shows a comparison plot of the experimental data and theoretical (model) curves obtained by integration of the kinetic model using the *n^th^* reaction model with average values obtained from Table 6: *n* = 1.4950 ± 0.0002, ln *A* = 27.5410 ± 0.0011. Regarding the autocatalytic model (actually a *n^th^* order model too), the average parameters used for the integration were: *n* = 1.4939 ± 0.0002, *m* = 0.0050 ± 0.0009, and ln *A* = 27.5654 ± 0.0009. As it can be seen, there is very good accuracy of the experimental data with both models (actually, the *n^th^* order reaction model since the autocatalytic model has a very low contribution of the autocatalytic component).

## 5. Conclusions

This work shows a systematic kinetic study of the thermal degradation of recycled ABS in nitrogen atmosphere. The obtained data by dynamic TGA at different heating rates, comprised between 2 and 30 K min^−1^, were used to apply different model-free kinetic methods (MFK) based on the isoconversional principle. With regard to the differential methods, the Friedman approach gave an *E_a_* value of 162.9 ± 1.3 kJ mol^−1^, which is quite similar to those obtained by applying several integral methods, such as FWO (*E_a_* = 163.4 ± 0.8kJ mol^−1^), KAS (*E_a_* = 163.0 ± 0.8 kJ mol^−1^), and the Starink method (*E_a_* = 163.3 ± 0.8 kJ mol^−1^). In addition, the variation of *E_a_* with the conversion α was almost negligible, thus suggesting the degradation process of ABS takes place in a single-step process. A different approach obtained by CKA gave an *E_a_* value of 163.7 ± 0.2 kJ mol^−1^, which is in total agreement with other isoconversional methods, thus giving clear evidence of the consistency of the CKA method. Once the consistency of the *E_a_* values was confirmed, the other kinetic parameters were obtained by calculating the y(α) and z(α) characteristic plots and comparing them with the master curve plots. Both y(α) and z(α) plots suggested a *n^th^* order reaction model. Then, both *n* and ln *A* were obtained by linear fitting resulting in *n* = 1.4950 ± 0.0002 and ln *A* = 27.5410 ± 0.0011 (*A* in min^−1^). Therefore, an excellent agreement between the experimental data and the values predicted by the kinetic model was found. In addition, the autocatalytic reaction model was tested, and the corresponding *n* and *m* exponents were: *n* = 1.4939 ± 0.0002, *m* = 0.0050 ± 0.0009, and ln *A* = 27.5654 ± 0.0009. These results indicated that the autocatalytic component has a very low contribution in the reaction model (extremely low *m* values) and it also corroborated the good agreement with a simple *n^th^* order reaction model.

## Figures and Tables

**Table 1:** Algebraic expressions for *g*(α) and *f*(α) for some of the mechanisms of solid state processes, activated by temperature.

Code	Reaction Model	*f(α)*	*g(α)*
P2	Power law	2α^1/2^	α^1/2^
P3	Power law	3α^2/3^	α^1/3^
P4	Power law	4α^3/4^	α^1/4^
A_2_	Nucleation and growth (Avrami Equation 1)	2(1−α)[−ln(1−α)]^1/2^	[−ln(1−α)]^1/2^
A_3_	Nucleation and growth (Avrami Equation 2)	3(1−α)[−ln(1−α)]^2/3^	[−ln(1−α)]^1/3^
A_4_	Nucleation and growth (Avrami Equation 3)	4(1−α)[−ln(1−α)]^3/4^	[−ln(1−α)]^1/4^
R_1_	Controlled reaction on the surface (motion in one dimension)	1	α
R_2_	Controlled surface reaction (contracting cylinder)	2(1−α)^1/2^	[−ln(1−α)]^1/2^
R_3_	Controlled reaction on the surface (migration volume; contracting sphere)	3(1−α)^2/3^	[−ln(1−α)]^1/3^
D_1_	Diffusion in one dimension	(1/2)α ^−1^	α ^2^
D_2_	Diffusion in two dimensions (Valensi Equation )	− [ln(1−α)]^−1^	(1−α) ln(1−α) + α
D_3_	Diffusion in three dimensions (Jander Equation )	(3/2)[1−(1−α)^1/3^]^−1^(1−α)^2/3^	[1 − (1−α)^1/3^]^2^
F_1_	Random nucleation with one nucleus of individual particle. Mample first order.	(1−α)	−ln(1−α)
F_2_	Random nucleation with two nuclei of individual particle	(1−α)^2^	(1−α)^−1^
F_3_	Random nucleation with three nuclei of individual particle	(1/2)(1−α)^3^	(1−α)^−2^
F_n_	*n^th^* order	(1−α)^n^	[(1−α)^1−*n*^−1]/(*n*−1)
ePT B_na_	Extended Prout-Tompkins	(1− α)^n^ α ^m^	No analytical solution

**Table 2:** Rational approximations for the temperature integral with different degrees.

*Degree*	*P(x)*
*1*	$\frac{e^{-x}}{x}\cdot \frac{1}{\left(x+2\right)}$
*2*	$\frac{e^{-x}}{x}\cdot \frac{\left(x+4\right)}{{(x}^{2}+6x+6)}$
*3*	$\frac{e^{-x}}{x}\cdot \frac{{(x}^{2}+10x+18)}{{(x}^{3}{+12x}^{2}+36x+24)}$
*4*	$\frac{e^{-x}}{x}\cdot \frac{{(x}^{3}{+18x}^{2}+86x+96)}{{(x}^{4}{+20x}^{3}+120x^{2}+240x+120)}$

**Table 3:** Summary of the thermal parameters corresponding to the temperature for the maximum degradation rate or peak maximum (*T_m_*), and their corresponding conversion (α*_m_*) and conversion rate (dα/d*T*)*_m_* values, for the thermal degradation of recycled ABS at different heating rates β.

Heating Rate β (K min^−1^)	*T_m_* (K)	α*_m_*	${\left(d\alpha /dT\right)}_{m}$
2	655	0.526	0.01454
5	675	0.535	0.01373
10	690	0.529	0.01314
15	699	0.525	0.01280
20	706	0.528	0.01256
25	711	0.524	0.01238
30	716	0.531	0.01223

**Table 4:** Summary of the main parameters of the Combined Kinetic Analysis (CKA) of the thermal degradation of recycled ABS as indicated in Equation (27).

Heating Rate β (K min^−1^)	*E_a_* (kJ mol^−1^)	ln *A c*	*r*	*n*	*m*
2	163.9	27.6810	−1.0000	1.4920	0.0024
5	163.9	27.6660	−1.0000	1.4930	0.0024
10	163.8	27.6510	−1.0000	1.4930	0.0024
15	163.5	27.5890	−1.0000	1.4920	0.0044
20	163.7	27.6220	−1.0000	1.4930	0.0032
25	163.6	27.6160	−1.0000	1.4930	0.0032
30	163.4	27.5810	−1.0000	1.4920	0.0044

**Table 5:** Experimental values corresponding to the maximum of the calculated y(α) and z(α) plots for the thermal degradation of recycled ABS at different heating rates.

Heating Rate β (K min^−1^)	$\alpha_{M}$	$\alpha_{p}^{\infty }$
2	0.0005	0.5547
5	0.0006	0.5621
10	0.0006	0.5556
15	0.0005	0.5635
20	0.0005	0.5333
25	0.0005	0.5611
30	0.0005	0.5557

**Table 6:** Main kinetic parameters obtained for the thermal degradation of recycled ABS by using the *n^th^* order (linear fit) and the autocatalytic (non-linear curve fit) reaction models obtained for different heating rates.

Heating Rate β (K min^−1^)	*n^th^* order: (1−*α*)^n^			autocatalytic: *α*^m^(1−*α*)^n^			
	*n*	ln *A*	*r*	*n*	*m*	ln *A*	*R* ^2^
2	1.4958	27.5423	0.9999	1.4929	0.0050	27.5659	1.000
5	1.4957	27.5419	1.0000	1.4935	0.0048	27.5655	1.000
10	1.4951	27.5411	1.0000	1.4938	0.0046	27.5649	1.000
15	1.4949	27.5408	1.0000	1.4939	0.0045	27.5646	1.000
20	1.4947	27.5405	1.0000	1.4941	0.0044	27.5644	1.000
25	1.4945	27.5403	1.0000	1.4942	0.0044	27.5643	1.000
30	1.4943	27.5402	1.0000	1.4952	0.0069	27.5684	1.000
